# Identification of Bacteria Utilizing Biphenyl, Benzoate, and Naphthalene in Long-Term Contaminated Soil

**DOI:** 10.1371/journal.pone.0040653

**Published:** 2012-07-13

**Authors:** Ondrej Uhlik, Jiri Wald, Michal Strejcek, Lucie Musilova, Jakub Ridl, Miluse Hroudova, Cestmir Vlcek, Erick Cardenas, Martina Mackova, Tomas Macek

**Affiliations:** 1 Department of Biochemistry and Microbiology, Faculty of Food and Biochemical Technology, Institute of Chemical Technology Prague, Prague, Czech Republic; 2 Department of Genomics and Bioinformatics, Institute of Molecular Genetics, Czech Academy of Sciences, Prague, Czech Republic; 3 Center for Microbial Ecology, Michigan State University, East Lansing, Michigan, United States of America; Argonne National Laboratory, United States of America

## Abstract

Bacteria were identified associated with biodegradation of aromatic pollutants biphenyl, benzoate, and naphthalene in a long-term polychlorinated biphenyl- and polyaromatic hydrocarbon-contaminated soil. In order to avoid biases of culture-based approaches, stable isotope probing was applied in combination with sequence analysis of 16 S rRNA gene pyrotags amplified from ^13^C-enriched DNA fractions. Special attention was paid to pyrosequencing data analysis in order to eliminate the errors caused by either generation of amplicons (random errors caused by DNA polymerase, formation of chimeric sequences) or sequencing itself. Therefore, sample DNA was amplified, sequenced, and analyzed along with the DNA of a mock community constructed out of 8 bacterial strains. This warranted that appropriate tools and parameters were chosen for sequence data processing. ^13^C-labeled metagenomes isolated after the incubation of soil samples with all three studied aromatics were largely dominated by Proteobacteria, namely sequences clustering with the genera *Rhodanobacter Burkholderia*, *Pandoraea*, *Dyella* as well as some *Rudaea*- and *Skermanella*-related ones. Pseudomonads were mostly labeled by ^13^C from naphthalene and benzoate. The results of this study show that many biphenyl/benzoate-assimilating bacteria derive carbon also from naphthalene, pointing out broader biodegradation abilities of some soil microbiota. The results also demonstrate that, in addition to traditionally isolated genera of degradative bacteria, yet-to-be cultured bacteria are important players in bioremediation. Overall, the study contributes to our understanding of biodegradation processes in contaminated soil. At the same time our results show the importance of sequencing and analyzing a mock community in order to more correctly process and analyze sequence data.

## Introduction

Wide-spread use, improper handling, and disposal of certain synthetic organic chemicals have resulted in contamination of soils, waters, and sediments. Among the compounds released, biphenyl, polychlorinated biphenyls (PCBs), petroleum chemicals including polycyclic aromatic hydrocarbons (PAHs) and BTEX (benzene, toluene, ethylbenzene and xylenes), pesticides, dioxins, furans, and flame retardants are of major concern [1], [2]. Their presence in the environment often poses a serious risk to both ecosystem functioning and human health. Although anthropogenic activity is a main source of these contaminants, they can as well form during natural events [3]. In addition, some biogenic sources of PAHs have been known, such as endogenic synthesis by microorganisms, phytoplankton, algae, or higher plants [4].

Although biphenyl, PCBs, and PAHs are toxic for many higher organisms, some bacteria are known to transform and/or mineralize these toxicants in the processes of bioremediation [4]–[6]. Under aerobic conditions, bacteria metabolize biphenyl and its monochlorinated derivatives [7], rarely also dichlorinated ones [8], in order to derive carbon and energy. Other lower chlorinated PCB congeners are usually biodegraded cometabolically [9]–[11] with biphenyl being the primary substrate. Degradative enzymes encoded in the *bph* operon transform (chloro)biphenyl to 2-hydroxypenta-2,4-dienoate and (chloro)benzoate. 2-hydroxypentadienoate is further metabolized into acetylcoenzyme A, which enters the Krebs cycle. (Chloro)benzoate is catabolized into catechol, which is further degraded into central intermediates [9].

The simplest PAH, naphthalene, along with some 3- and 4-ring PAHs act as growth substrates for aerobic bacteria [12]. Higher PAHs are usually biodegraded cometabolically and their biotransformation yields no carbon or energy [13]. The upper catabolic pathway for naphthalene degradation consists of six steps with salicylic acid being the final product. Salicylic acid is further metabolized via catechol or gentisic acid by so called lower pathway of naphthalene degradation. In many cases, the same enzymes can transform not only naphthalene but also phenanthrene and anthracene [12].

In order to understand natural biodegradative processes in contaminated environments, it is crucial to identify bacteria involved in pollutants metabolism. Most of our knowledge on biodegradative processes in soil has still been based on highly biased results of cultivation studies. Stable isotope probing (SIP) [14], in contrast, enables researchers to link metabolic activity and phylogeny without previous cultivation of microbes. Therefore, SIP is considered one of the leading molecular tools for investigating the diversity of bacteria potentially responsible for ecologically relevant processes. Applying this method first helped describe the utilization of one-carbon compounds [e.g. 14–16] and has since been used to detect microbial communities active in the utilization of a wide variety of compounds, including xenobiotics [reviewed by 17,18]. Huge progress in application of SIP has been made as a result of its combination with high-throughput sequencing [19]. For the purposes of bacterial identification, the most informative approach appears to be pyrosequencing of 16 S rRNA tag-encoded amplicons. Recently, combination of SIP with sequence analysis of 16 S rRNA gene pyrotags has been used to probe populations associated with rapid response to soil rewetting [20], nitrification [21], nitrogen incorporation in petroleum-contaminated arctic soils [22], earthworm-mediated shaping of communities [23], toluene degradation at a tar-oil-contaminated aquifer [24], or biphenyl-oxidation in a tidal mudflat [25]. At the same time, bottlenecks of this approach have been realized which are associated especially with the noise introduced during amplification and pyrosequencing leading to an overestimation of diversity [26]. Such a realization has resulted in the introduction of several denoising algorithms or improved operational taxonomic unit (OTU) clustering [27]–[30] in order to increase data analysis exactness.

The aim of the presented study was to identify bacterial populations in contaminated soil that derive carbon from biphenyl, naphthalene, and benzoate. In order to reach this goal, we applied DNA-based stable isotope probing with subsequent pyrosequencing of 16 S rRNA gene tag-encoded amplicons. Sequence data were analyzed along with a mock community comprised of known strains in order to determine appropriate parameters for sequence data processing. Results of the study help unveil which bacteria are potentially involved in biodegradation of aromatic pollutants. In addition, the experimental design with labeled substrates as well as key intermediates of degradation pathways (biphenyl and benzoate, respectively) helps clarify the flow of carbon in the contaminated soil. The experimental design also has another advantage that it can detect abilities of strains to biodegrade multiple contaminants.

## Results

### Stable Isotope Probing of DNA

Microcosms constructed with contaminated soil and amended with ^13^C-labeled biphenyl, benzoate, or naphthalene were destructively harvested after 4 and 14 days of incubation. Majority of unlabeled DNA occurred at buoyant density of around 1.6 g.mL^–1^. Labeled DNA was localized in fractions with buoyant densities 1.623–1.673 g.mL^–1^ as is indicated by comparing the distribution of 16 S rRNA genes in gradient fractions of unlabeled control and ^13^C-DNA. These fractions were compiled and further analyzed as ^13^C-DNA (“heavy” DNA). One of the major issues associated with stable isotope probing is to control for background ^12^C-DNA contamination in the heavy-DNA-containing fractions. To cope with this phenomenon, fractions of unlabeled control with buoyant densities of 1.623–1.673 g.mL^–1^ were compiled and further analyzed along with the samples. The sequences from all samples that were ≥99% identical to those of unlabeled control were subtracted from analysis as they were considered background ^12^C-DNA contamination.

Distributions of 16 S rRNA genes in gradient fractions indicate that in case of biphenyl significant labeling was achieved on day 14, whereas on day 4 DNA was not labeled enough to be distinguished from the unlabeled control (the curves have almost the same behavior). Incubation time of 4 days, however, was sufficient for cells to derive carbon from ^13^C-labeled benzoate and naphthalene (Figure S1).

### Identification of Bacteria

**Figure 1:**
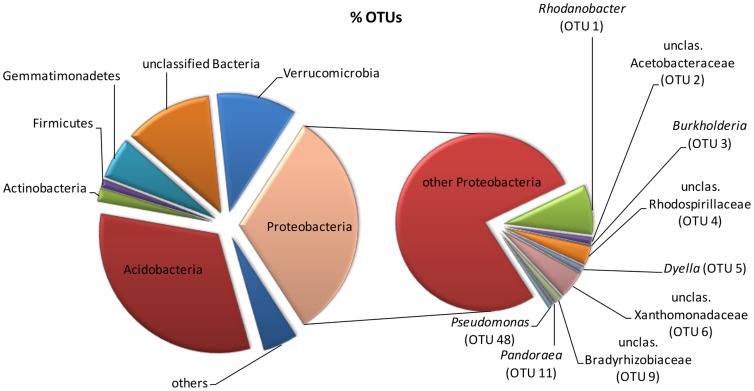
Phylogenetic identification (at the phylum level) of sequences retrieved from soil total community with special focus on metabolically active Proteobacteria (OTUs are described in Table S1).

The majority of 16 S rRNA gene sequences retrieved from the contaminated soil clustered with Proteobacteria and Acidobacteria – together these two phyla represented two thirds of the sequences (Figure 1). Sequences of the phylum Proteobacteria were also the predominant ones retrieved from ^13^C-DNA. ^13^C-DNA obtained after a 4-day incubation with ^13^C-biphenyl contained less than 25% of valid sequences (Table 1). This was caused by subtracting the majority of sequences that were also detected in the unlabeled control. Such a small number of sequences was expected based on data obtained from qPCR (Figure S1), which suggested there was not enough DNA labeling on day 4. Analyzing ^13^C-DNA isolated after 14-day incubation of the soil with biphenyl showed that carbon from biphenyl has been mainly utilized by the genera *Rhodanobacter* and *Burkholderia* (38% of all sequences) (Table 2). The major OTU identified as *Rhodanobacter* was most likely associated with *R. spathiphylli* as determined by RDP Seqmatch. In addition to *Rhodanobacter*, other Gammaproteobacteria from the family Xanthomonadaceae have been detected, including *Dyella* and *Rudaea*-related populations. Betaproteobacteria were represented in addition to *Burkholderia* by *Pandoraea*. Further bacteria included in the top OTUs belonged to Alphaproteobacteria (Table 2). Many of the sequences clustering with major OTUs were not classified at the level of genus, suggesting a possible detection of yet-to-be described bacterial species.

**Table 1:** Number of sequences obtained after sequence processing, after subtracting sequences detected also in control DNA (valid sequences), and after normalizing.

Sample	Sequences after processing	Valid sequences	Normalized sequences
**Total community**	31388	31388	244
**Bp4**	1479	343	343
**Bp14**	23189	22610	329
**Bz4**	5314	4750	327
**Bz14**	25029	21485	305
**Np4**	20721	19625	332
**Np14**	8029	4685	336

The majority of sequences detected after ^13^C-benzoate labeling clustered with pseudomonads. This OTU was also detected in ^13^C-biphenyl labeled DNA on day 14 but was very scarce (0.3% sequences). The second most abundant cluster of sequences after both 4- and 14-day incubation was identical to the *Rhodanobacter* cluster which dominated carbon acquisition from ^13^C-biphenyl. Similarly, other populations were detected to derive carbon from benzoate as well as biphenyl, including those associated with *Burkholderia*, *Pandoraea*, or *Rudaea*-like and *Skermanella*-like Proteobacteria, and some Acidobacteria (Table 2, Table S1). In addition, carbon from ^13^C-benzoate was acquired by populations of *Azotobacter* (3% and 6% sequences on day 4 and 14, respectively) and Gram-positive bacilli (2% and 7% sequences on day 4 and 14, respectively) (Table 2).

**Table 2:** Top OTUs detected in ^13^C-DNA after incubation of soil with ^13^C-biphenyl, ^13^C-benzoate, and ^13^C-naphthalene.

	% of seq^a^	Identification^b^	Closest type strain(s)^c^	score^d^	OTU co-occurence^e^
**biphenyl, 14 days**	28	*Rhodanobacter*	*R. spathiphylli* B39; AM087226	1.000	Bz4, Bz14, Np4, Np14
	10	*Burkholderia*	*B. xenovorans* LB400; U86373 *B*	1.000	Bz4, Bz14, Np4, Np14
			. *caledonica* LMG 19076; AF215704		
			*B. ginsengisoli* KMY03; AB201286		
			*B. phytofirmans* PsJN; AY497470		
			*B. megapolitana* LMG 23650; AM489502		
	6	unclassified Acetobacteraceae	*Acidicaldus organivorans* Y008; AY140238	0.686	Bz4, Bz14, Np14
	5	unclassified Rhodospirillaceae	*Skermanella xinjiangensis* 10-1-101; EU586202	0.750	Bz4, Bz14, Np4, Np14
	4	*Dyella*	*D. ginsengisoli* Gsoil 3046; AB245367	1.000	Bz14, Np4
	3	unclassified Xanthomonadaceae	*Rudaea cellulosilytica* KIS3-4; EU741687	0.815	Bz4, Bz14
	2	unclassified Bradyrhizobiaceae	*Bradyrhizobium* spp. (more type strains)	1.000	Bz4, Bz14
			*Afipia broomeae* F186; U87759		
			*Agromonas oligotrophica* JCM 1494; D78366		
	2	*Pandoraea*	*P. apista* LMG 16407; AF139173	1.000	Bp4, Bz4, Bz14, Np4
			*P. pulmonicola* LMG 18106; AF139175		
			*P. pnomenusa* CCUG 38742; AY268170		
	2	unclassified Bacteria	*Desmospora activa* IMMIB L-1269; AM940019	0.486	Bz4, Bz14
**benzoate, 4 days**	25	*Pseudomonas*	*P. umsongensis* Ps 3-10; AF468450	1.000	Bz14, Bp4, Bp14, Np4, Np14
			*P. mandelii* CIP 105273; AF058286		
			*P. migulae* CIP 105470; AF074383		
			*P. reinekei* MT1; AM293565		
			*P. arsenicoxydans* VC-1; FN645213		
	9	*Rhodanobacter*	*R. spathiphylli* B39; AM087226	1.000	Bz14, Bp14, Np4, Np14
	6	*Gemmatimonas*	*G. aurantiaca* T-27; AB072735	0.570	Np14
	3	unclassified Bacteria	*Desmospora activa* IMMIB L-1269; AM940019	0.486	Bz14, Bp14
	3	*Sphingomonas*	*S. mali* IFO 10550-T; Y09638	1.000	Np14
			*S. asaccharolytica* IFO 15499-T; Y09639		
			*S. melonis* PG-224; AB055863		
			*S. aquatilis* JSS-7; AF131295		
			*S. panni* C52; AJ575818		
			*S. hankookensis* ODN7; FJ194436		
	3	unclassified Rhodospirillaceae	*Skermanella xinjiangensis* 10-1-101; EU586202	0.750	Bz14, Bp14, Np4, Np14
	3	*Azotobacter*	*Pseudomonas azotifigens* 6H33b; AB189452	0.943	Bz14
			*A. chroococcum* IAM 12666; AB175653	0.935	
	2	*Sporosarcina*	*Bacillus* spp. (more type strains)	1.000	Bz14
			*S. antarctica* N-05; EF154512	0.963	
	2	unclassified Xanthomonadaceae	*Rudaea cellulosilytica* KIS3-4; EU741687	0.815	Bz14, Bp14
**benzoate, 14 days**	28	*Pseudomonas*	*P. umsongensis* Ps 3-10; AF468450	1.000	Bz4, Bp4, Bp14, Np4, Np14
			*P. mandelii* CIP 105273; AF058286		
			*P. migulae* CIP 105470; AF074383		
			*P. reinekei* MT1; AM293565		
			*P. arsenicoxydans* VC-1; FN645213		
	10	*Rhodanobacter*	*R. spathiphylli* B39; AM087226	1.000	Bz4, Bp14, Np4, Np14
	7	*Sporosarcina*	*Bacillus* spp. (more type strains)	1.000	Bz4
			*S. antarctica* N-05; EF154512	0.963	
	6	*Azotobacter*	*Pseudomonas azotifigens* 6H33b; AB189452	0.943	Bz4
			*A. chroococcum* IAM 12666; AB175653	0.935	
	4	unclassified Xanthomonadaceae	*Rudaea cellulosilytica* KIS3-4; EU741687	0.815	Bz4, Bp14
	3	*Burkholderia*	*B. xenovorans* LB400; U86373 *B*	1.000	Bz4, Bp14, Np4, Np14
			. *caledonica* LMG 19076; AF215704		
			*B. ginsengisoli* KMY03; AB201286		
			*B. phytofirmans* PsJN; AY497470		
			*B. megapolitana* LMG 23650; AM489502		
	3	unclassified Bacteria	*Desmospora activa* IMMIB L-1269; AM940019	0.486	Bz4, Bp14
	2	*Pandoraea*	*P. apista* LMG 16407; AF139173	1.000	Bz4, Bp4, Bp14, Np4
			*P. pulmonicola* LMG 18106; AF139175		
			*P. pnomenusa* CCUG 38742; AY268170		
	2	unclassified Acetobacteraceae	*Inquilinus limosus* AU476; AY043374	0.700	Bz4, Bp14
			*Inquilinus ginsengisoli* Gsoil 080; AB245352		
	2	unclassified Rhodospirillaceae	*Skermanella xinjiangensis* 10-1-101; EU586202	0.750	Bz4, Bp14, Np4, Np14
**naphthalene, 4 days**	81	*Pseudomonas*	*P. umsongensis* Ps 3–10; AF468450	1.000	Np14, Bp4, Bp14, Bz4, Bz14
			*P. mandelii* CIP 105273; AF058286		
			*P. migulae* CIP 105470; AF074383		
			*P. reinekei* MT1; AM293565		
			*P. arsenicoxydans* VC-1; FN645213		
	8	*Rhodanobacter*	*R. spathiphylli* B39; AM087226	1.000	Np14, Bp14, Bz4, Bz14
	2	unclassified Rhodospirillaceae	*Oceanibaculum pacificum* MC2UP-L3; FJ463255	0.703	Bz14
	2	Acidobacteria Gp6	*Ruminobacter amylophilus* DSM 1361; Y15992	0.510	Bp14, Bz4, Bz14
	2	*Dongia*	*D. mobilis* LM22; FJ455532	0.844	Bp14, Bz14
	1	unclassified Proteobacteria	*Caloramator fervidus* RT4.B1; L09187	0.541	Np14
	1	unclassified Rhodospirillaceae	*Skermanella xinjiangensis* 10-1-101; EU586202	0.750	Np14, Bp14, Bz4, Bz14
	1	*Pandoraea*	*P. apista* LMG 16407; AF139173	1.000	Bp4, Bp14, Bz4, Bz14
			*P. pulmonicola* LMG 18106; AF139175		
			*P. pnomenusa* CCUG 38742; AY268170		
	1	*Propionibacterium*	*P. granulosum* DSM 20700; AJ003057	1.000	Np14
	1	*Aquicella*	*A. lusitana* SGT-39; AY359282	0.663	–
**naphthalene, 14 days**	40	*Pseudomonas*	*P. umsongensis* Ps 3–10; AF468450	1.000	Np4, Bp4, Bp14, Bz4, Bz14
			*P. mandelii* CIP 105273; AF058286		
			*P. migulae* CIP 105470; AF074383		
			*P. reinekei* MT1; AM293565		
			*P. arsenicoxydans* VC-1; FN645213		
	15	*Rhodanobacter*	*R. spathiphylli* B39; AM087226	1.000	Np4, Bp14, Bz4, Bz14
	8	unclassified Acetobacteraceae	*Roseomonas stagni* HS-69; AB369258	0.734	–
			*R. frigidaquae* CW67; EU290160		
	7	unclassified Rhodospirillaceae	*Skermanella xinjiangensis* 10-1-101; EU586202	0.750	Np4, Bp14, Bz4, Bz14
	5	*Aeromicrobium*	*A. marinum* T2; AY166703	1.000	–
			*A. ginsengisoli* Gsoil 098; AB245394		
			*A. erythreum* NRRL B-3381; AF005021		
			*A. halocynthiae* KME 001; FJ042789		
	4	unclassified Proteobacteria	*Caloramator fervidus* RT4.B1; L09187	0.541	Np4
	4	unclassified Bacteria	*Calditerricola yamamurae* YMO722; AB308475	0.551	Bp14, Bz4
	2	Verrucomicrobia Subdiv. 3	uncultured Verrucomicrobium DEV008; AJ401115	0.611	–
	2	*Propionibacterium*	*P. granulosum* DSM 20700; AJ003057	1.000	Np4
	2	*Gemmatimonas*	*G. aurantiaca* T-27; AB072735	0.570	Bz4

Identification was performed by mothur-implemented RDP reference files [78] and the closest type strain was determined by RDP Seqmatch with the representative sequence of each OTU [76]. The entire dataset is in Table S1.

a Relative abundance of sequences.

b Identification of OTU based on identification of the representative at the level of genus as determined by RDP classifier (using 50% threshold).

c Determined by RDP Seqmatch.

d Score represents S_ab_ score – the number of (unique) 7-base oligomers shared between the sequence data and a given RDP sequence divided by the lowest number of unique oligos in either of the two sequences.

e Refers to samples where the same OTU was detected.

Sequences detected in ^13^C-DNA isolated from ^13^C-naphthalene-amended microcosms were also largely predominated by pseudomonads. Their predominance is especially obvious on day 4. On day 14, the peak representing heavy DNA was much smaller compared to day 4 (Figure S1) and also many sequences detected in heavy DNA on day 14 were classified as ^12^C-background (Table 1). The major cluster of pseudomonads corresponds to the major ones detected in benzoate-amended microcosms, i.e. identical populations of pseudomonads seem to acquire carbon from both, benzoate and naphthalene. Many other sequences detected in ^13^C-DNA after naphthalene labeling were identical to those detected after biphenyl and benzoate labeling, including *Rhodanobacter*, *Burkholderia*, *Pandoraea*, *Dyella* as well as many OTUs not classified at the level of genus. These populations thus seem to play a key role in intrinsic bioremediation of a wider variety of aromatic contaminants in the soil.

### Mock Community Analysis

Pyrosequencing of 16 S rRNA genes of 8 strains included in our mock community was performed in order to (i) analyze the accuracy and efficiency of the amplification and sequencing strategy and (ii) determine appropriate parameters for sequence data processing. A mock community was constructed with strains from phyla Proteobacteria, Actinobacteria and Firmicutes (Table 3). Members of these phyla are most commonly associated with the degradation of aromatics. From over 40,000 reads obtained by pyrosequencing, a random subset of 1,000 and 10,000 sequences was chosen which was further used as a testing set for determining the appropriate analysis procedures. Details of the analysis performed in mothur software package, version 1.25 [31] are described in *Material and methods* section.

**Table 3:** Bacterial strains used for the preparation of the mock community.

Bacterium	BioProjectAccession	Reference
*Achromobacter xylosoxidans* A8	PRJNA59899	[79]
*Pseudomonas putida* JB	to be released	[35]
*Rhodobacter capsulatus* SB 1003	PRJNA47509	[80]
*Agrobacterium tumefaciens* C58	PRJNA57865	[81]
*Arthrobacter chlorophenolicus* A6	PRJNA58969	[82]
*Bacillus pumilus* SAFR-032	PRJNA59017	[83]
*Micrococcus luteus* NCTC 2665	PRJNA59033	[84]
*Rhodococcus jostii* RHA1	PRJNA58325	[85]

The number of OTUs (defined at 3% distance) detected in the subset differed depending on the program used to detect and remove putative chimeric sequences. Using UCHIME [32] for checking chimeras, the processing resulted in 19 OTUs (defined at 3% distance) whereas using Perseus [29], the final number of OTUs was reduced to 13. The same trend was obvious when a subset of 10,000 reads of mock community were analyzed – using UCHIME and Perseus, the total number of OTUs was 64 and 41. After OTUs represented by 3 or less sequences were subtracted, the numbers of OTUs were reduced to 14 and 8, respectively. Applying Perseus, therefore, resulted in the expected number of OTUs (i.e. 8) after the exclusion of tripletons, doubletons, and singletons. After normalizing sequence data, the spurious OTUs were restricted to singletons only (Table S1).

Other algorithms for chimera checking, such as Bellerophon [33] or B2C2 [34], which is a stand-alone software not implemented in mothur, had a worse performance than both Perseus and UCHIME (data not shown). Therefore, sequence data were processed according to commands described in Table S2 with the use of Perseus for identifying chimeric sequences.

## Discussion

In order to better understand, optimize and/or monitor bioremediation processes, a fundamental goal has been to identify bacteria involved in biodegradation of pollutants. The aim of this study was to investigate which bacterial populations participate in biphenyl, benzoate, and naphthalene biodegradation in real long term contaminated soil. This soil represents a unique ecosystem since it was gradually contaminated for 30 years and it has been deposited at the present location for 15 years [35]. Such a long time should have been sufficient for the establishment of stable microbial populations. Cultivation-based techniques highly underestimate diversity as only 1% of microbes are routinely cultivated under laboratory conditions [36], [37]. Linking contaminant transformation to phylogenetic identity of active microbes without cultivation has thus become the main challenge. SIP is one of the available methods of functional molecular microbial ecology which can meet this challenge [38]. In this study, SIP was combined with pyrosequencing of 16 S rRNA gene amplicons. Such an approach should warrant diversity analysis in sufficient depth.

The results of stable isotope probing experiments show that biotransformation of aromatic pollutants in this soil is mainly mediated by Proteobacteria There were populations detected that derived carbon from both labeled biphenyl and benzoate, including *Rhodanobacter*, *Burkholderia*, *Pandoraea*, *Dyella*, as well as *Rudaea*- and *Skermanella*-related bacteria. As a sufficient amount of labeled DNA was not achieved on day 4 compared to unlabeled control, the question remains whether ^13^C from biphenyl was derived either directly by biphenyl degradation or indirectly via cross-feeding (possibly with benzoate as a substrate). If the latter was the case, one would expect similar distribution of sequences in ^13^C-DNA isolated after the incubation with both labeled biphenyl and benzoate. However, the richest clusters in ^13^C-DNA after labeling with benzoate were associated with pseudomonads (Table 2) whereas in ^13^C-DNA after labeling with biphenyl, pseudomonads were much poorer in abundance (Table S1). Therefore, it seems to be more likely that the populations detected are capable of transforming biphenyl via benzoate into compounds of intermediary metabolism. This may as well be supported by previous reports showing that members of majority of these genera isolated in pure cultures are capable of degrading biphenyl via benzoate to Krebs cycle intermediates. These include some of the model PCB degrading strains *Burkholderia xenovorans* LB400 [39], [40] or *Pandoraea pnomenusa* (formerly *Comamonas testosteroni*) B-356 [41], [42]. Recently also the closest type strain to the *Dyella* cluster, *D. ginsengisoli*, has been described to degrade biphenyl [43]. The question remained whether such populations can be metabolically active directly in the soil, which has been confirmed by our results. In previous SIP experiments, however, only *Burkholderia* members were found to be actively deriving carbon from biphenyl [44] and benzoate [45], [46] in soils. All other dominant populations acquiring carbon from biphenyl and benzoate directly in this soil are reported here for the first time. In addition, based on cultivation studies *Rhodanobacter* members were previously associated only with the degradation of PAHs [47], chlorobenzoates [48], or certain pesticides [49]. This paper, to the best of our knowledge, is the first one to report bacteria clustering with *Rhodanobacter* spp. to derive carbon from biphenyl. However, there have been studies published detecting *Rhodanobacter* spp. in total community DNA isolated from PCB-contaminated soils [50], [51].

Pseudomonads, which have been reported to be the most prevailing group of bacteria to degrade complex organic compounds [52], largely dominated carbon acquisition from labeled benzoate and naphthalene in this study. A much lower number of sequences associated with this genus were detected to have derived carbon from biphenyl. Many strains belonging to pseudomonads had been reported to degrade biphenyl and PCBs [53]–[56], PAHs [12], or other aromatic contaminants. *Pseudomonas* spp. were also detected in other SIP experiments to utilize (i) naphthalene in PAH-contaminated soil [57]–[59], aquifer [60], and an aerobic bioreactor used to treat contaminated soil [61]; (ii) benzoate in soil [46]; and (iii) biphenyl in PCB-contaminated river sediment [62] or soil [63]. Major OTUs detected in ^13^C-DNAs were also largely detected in the total community DNA (Figure 1). In addition, the OTU associated with *Rhodanobacter spathiphylli* was the most abundant taxon of all (Table S1). Therefore, these populations do not seem to be just opportunistic growers taking advantage of external substrates they were provided with during SIP but are rather regular abundant members in this contaminated soil adapted for the biodegradation of the pollutants.

Other metabolically active Proteobacteria included *Azotobacter*, *Sphingomonas*, and unclassified Rhodospirillaceae, whose sequences were retrieved from ^13^C-DNA isolated after incubation with ^13^C-labeled benzoate only, or benzoate and naphthalene, or all three studied compounds, respectively. A relatively large proportion of sequences retrieved from ^13^C-DNA were not classified at the level of genus. These can be either sequences of bacteria with no cultured and studied representatives or chimeric sequences that were not removed by Perseus. The fact the same OTUs are often present in more samples (Table 2) suggests the former to be much more likely.

In addition to Proteobacteria, bacteria lineages of Gemmatimonadetes, Acidobacteria, and Gram-positive bacilli were detected to derive carbon from some of the labeled aromatics. Ability to degrade several organic pollutants have already been demonstrated for genera *Azotobacter*, *Sphingomonas*, *Bacillus*, and *Gemmatimonas* [64]–[67], with *Bacillus* and *Sphingomonas* being detected by SIP experiments as a benzoate [46] and biphenyl and pyrene [63], [68], [69] utilizers, respectively.

Contaminated soil from the locality of Lhenice has been subjected to certain phyto/rhizoremediation studies previously, including isolation of biphenyl (and potentially PCB) degrading bacteria. Only isolates of the genus *Achromobacter*, however, were identified in this study to be metabolically active in biphenyl biotransformation (Table S1, OTU 64) and were previously obtained in pure culture [70]. Although Ionescu et al. [71] isolated a *Burkholderia* member, the identification was based on biochemical characteristics; 16 S rRNA sequencing showed this strain to cluster with the genus *Achromobacter* [35]. In addition, a time-course stable isotope probing experiment was conducted with the soil from this locality detecting *Hydrogenophaga* and *Paenibacillus* as dominant biphenyl-metabolizing genera. Yet the microcosms were constructed by converting the soil into slurry and enriching biphenyl utilizers prior to stable isotope probing [50]. These factors can create favorable conditions for different microbial populations, which we avoided in this study. This is in agreement with results of another study [25], in which *Paenibacillus* sequences were also found to dominate ^13^C-biphenyl-labeled DNA in tidal mudflat.

It was previously highlighted that pyrosequencing of amplicons can overestimate microbial diversity and the datasets need qualified processing before any conclusions are made [26]. This can be performed, for instance, through denoising pyrosequencing amplicon reads by exploiting rank-abundance distributions [28], through denoising flowgrams before these are converted to sequences [29], [30], or through improved OTU clustering [27]. Many of these studies also highlight the need to remove chimeric sequences in order to accurately estimate the diversity of a sample. In our study, we applied the mock community analysis-based approach for optimization of data processing in mothur software package (Table S2). Our data processing was based on standardized operating procedure defined for the needs of Human Microbiome Project [72], which uses UCHIME to identify chimeric sequences. Based on our mock community processing Perseus performed even better. Therefore, it was applied when processing sequence data.

Overall, our data show that biotransformation of selected aromatics was mainly performed by the same populations of Proteobacteria. Stable isotope probing combined with ultra-deep sequencing is shown here to be a helpful tool to monitor natural attenuation and investigate bioremediation potential. Results obtained within this study bring insight into the identity of bacteria actively metabolizing contaminants in soil. It points out broader biodegradation abilities of some soil microbiota, which can be further employed in bioremediation of sites with mixed organic contaminants. Many of the metabolically active populations are as-yet uncultured and unclassified bacteria. Therefore, this study shows the importance of cultivation-independent techniques to investigate biodegradation processes. At the same time our results show the importance of sequencing and analyzing a mock community in order to more correctly process and analyze sequence data.

## Materials and Methods

### SIP Microcosms

The source of soil samples was a dumpsite of contaminated soil in Lhenice, south Bohemia, Czech Republic [73]. The soil originated from a tarmacadam-producing plant in Milevsko, South Bohemia, where it was gradually contaminated from 1960s till 1990. In 1996, the soil was transferred and deposited at the present location. The main contaminants are PCBs. Their total amounts have decreased over the years by natural attenuation, from the firstly determined concentration of 470 µg PCBs/g soil in 1999 [35] to about 100 µg PCBs/g soil in 2008. The sum of PCBs was based solely on indicator congeners so the actual numbers of all congeners are even higher. In addition to PCBs, the soil contains biphenyl, PAHs, pesticides (DDT, traces of hexachlorbenzene and lindane), and heavy metals (Table 4).

**Table 4:** Analysis of inorganic nutrients and contaminants available in the soil.

Parameter	Unit	Result	Standard error
inorganic carbon	% dry matter^a^	1.46	11%
inorganic nitrogen		0.12	55%
inorganic sulfur		<0.1	–
sum of PCBs 28, 52, 101, 118, 138, 153, 180	mg/kg dry matter^b^	96.74	40%
sum of carcinogenic PAHs	mg/kg dry matter^c^	0.33	30%
sum of non-carcinogenic PAHs		0.30	30%
Fe	mg/kg dry matter^d^	33,860	20%
Ni		51.9	20%
Zn		88.0	20%

Results shown are averages from 5 independently measured samples (performed commercially).

a Based on method CZ_SOP_D06_07_121.

b Based on method US EPA 8082.

c Based on methods EPA 8270, EPA 8131, EPA 8091.

d Based on method US EPA 200.7.

The soil samples for the experiment were collected from the depth of about 0.5 m in the late summer of 2008. After coarse-sieving and homogenization, a subsample of 60 g was fine-sieved and transferred on a slide of filter-paper in a box with a perforated bottom. The box was closed and inserted into another box with water on the bottom so that the soil was not in contact with the liquid. In such arrangement, the soil was left to moisturize for 3 days in order to increase bacterial activity. SIP microcosms were set in 100 mL serum bottles (Sigma-Aldrich, USA) in triplicates, each containing 2.5 g soil. ^13^C-labeled biphenyl and naphthalene (Sigma-Aldrich, USA) were dissolved in acetone (concentration 50 mg.mL^–1^), and 10 µL of the solutions were applied onto the inner walls of empty serum bottles. The soil was added after acetone was completely evaporated leaving behind crystals of the substrates. Subsequently, the soil was moisturized with 500 µL of mineral salt solution [50]. Bottles were sealed with crimp seals with silicone septa to maintain stable atmosphere with continuous evaporation of the substrates at the beginning of the incubation. ^13^C-labeled benzoate (Sigma-Aldrich, USA) was applied directly in mineral salt solution (500 µL, concentration 1 mg/mL) onto the soil. Microcosms were destructively harvested by freezing (−80°C) after 4 and 14 days of incubation at 20°C. In order to minimize a possible influence of soil heterogeneity, triplicate SIP microcosms were set for each substrate and each time point. After the incubation period, triplicate samples were pooled for downstream processes to recover ^13^C-DNA from all three replicates at the same time.

### ^13^C-DNA Isolation

DNA was extracted with a PowerMax Soil DNA Isolation Kit (MoBio Laboratories Inc., USA) using the standard protocol. After final elution, DNA was concentrated by ethanol precipitation with glycogen (Roche, Germany) as described previously [50]. Pellets were dissolved in 50 µL of molecular biology water (Sigma-Aldrich, USA), and samples were diluted to a concentration of 100 ng.µL^–1^. In 2 mL centrifugation cuvettes, 8 µL of DNA solution (800 ng DNA) was mixed with cesium trifluoroacetate (Amersham, UK) adjusted to a density of 1.6 g.mL^–1^. Isopycnic centrifugation was performed on a Discovery 90 Ultracentrifuge using TFT-80.2 Fixed-Angle Ultraspeed Centrifuge Rotor (Sorvall, USA) at conditions of 145,000×*g* for 70 hours in 2 mL cuvettes. Using a Beckman Fraction Recovery System (Beckman Coulter, USA) and Harvard Pump 11 Plus Single Syringe (Harvard Apparatus, USA), each gradient was fractionated into 50 µL fractions (with flow rate 200 µL.min^−1^). Buoyant density of each fraction was determined based on refractive index of fractionated blanks (water was used instead of DNA) measured with Digital Handheld Refractometer (Reichert Analytical Instruments, USA). DNA from fractions with buoyant densities of 1.559–1.697 g.mL^–1^ was retrieved by isopropanol precipitation with glycogen [50]. Quantification of 16 S rRNA genes in these fractions was carried out using real-time qPCR in relation to a standard curve constructed with *Pseudomonas stutzeri* JM300 genomic DNA, which contains 213,000 16 S rRNA gene copies per ng of genomic DNA [74]. PCR conditions were as follows: each 12 µL reaction contained 1× DyNAmo Flash SYBR Green qPCR Mastermix (Finnzymes, Finland), 4 pmol of each primer 786f, 5′-GATTAGATACCCTGGTAG-3′, and 939r, 5′-CTTGTGCGGGCCCCCGTCAATTC-3′ [75], and 2 µL of template DNA from each fraction. Cycling program was set to 95°C for 5 min, 35 cycles of 95°C for 20 s, 55°C for 30 s, 72°C for 30 s, and a final extension at 72°C for 10 min. Each measurement was performed in duplicates.

### Amplicon Preparation and Pyrosequencing

Regions V4–V5 of 16 S rRNA genes were amplified with primers (numbering according to *E. coli* [J01695] positioning) f563–577, 5′-AYTGGGYDTAAAGNG-3′, and r926–909, 5′-CCGTCAATTCMTTTRAGT-3′ (www.rdp.cme.msu.edu) [76]. Each of the primers was synthesized together with sequencing adapters (454 Sequencing Application Brief No. 001-2009, Roche), and the forward primer was also modified with different tags (454 Sequencing Technical Bulletin No. 005-2009, Roche) so that more samples could be pooled and sequenced at once.

The PCR mixture was prepared in 12.5 µL volumes containing FastStart High Fidelity Reaction Buffer (Roche, Germany), 2.5 nmol of dNTPs, 2.5 pmol of each primer (Generi Biotech, Czech Republic), 1.125 µg bovine serum albumin (New England BioLabs, Great Britain), 0.625 U of FastStart High Fidelity PCR System Enzyme Blend (Roche Diagnostics) and template DNA (5–50 ng). The reaction conditions were as follows: 95°C for 5 min, 35 cycles of 95°C for 20 s, 52°C for 30 s, and 72°C for 60 s with final extension at 72°C for 10 min. Obtained PCR products were used as templates for reconditioning PCR [77] in order to minimize non-specific products. Reconditioning PCR was performed in 50 µL volumes using the same PCR reagent concentrations as described above and 5 µL of template DNA. The cycling conditions were the same except the number of cycles was between 5 and 8. PCR products were purified with a QIAquick PCR purification kit (Qiagen, Germany) and mixed together with other barcoded samples for pyrosequencing. The amplicon pool was purified using AMPure XP Beads (Agencourt, Beckman Coulter, USA) to remove residual primer-dimers according to manufacturer’s instructions. Amplicons were unidirectionally sequenced from the forward primer using GS FLX Titanium chemistry followed by amplicon analysis of signal processing (Roche).

### Mock Community as a Control for Pyrosequencing

A mock community was constructed by mixing genomic DNA of eight bacterial strains (Table 3). After the strains were grown overnight in liquid Luria Bertani medium (Oxoid, UK), their genomic DNA was isolated with a PureLink Genomic DNA Mini Kit (Invitrogen, USA). The number of 16 S rRNA gene copies per ng of each genomic DNA was determined by real-time qPCR as described above. Genomic DNA samples of the selected strains were mixed so that the numbers of 16 S rRNA genes from each strain were of the same order of magnitude. Only the amount of *Rhodococcus jostii* RHA1 16 S rRNA genes was an order of magnitude lower.

### Analysis of 16 S rRNA Pyrotags

Raw pyrosequencing data (*.sff files) were processed within mothur software package, version 1.25 [31]. Except some minor modifications, processing was based on standardized operating procedure defined for the needs of Human Microbiome Project [72]. The whole procedure is described in Table S2 and was set this way based on mock community (Table 3) analysis. Sequences in ^13^C-DNA which were more than 99% identical to those detected in control set (DNA from fractions of unlabeled control corresponding to those where ^13^C-DNA was detected in samples after probing) were considered contamination and were subtracted from analyses.

Sequences with less than 3% sequence similarity were considered the same operational taxonomic units (OTUs). These were classified by mothur-implemented RDP reference files [78] and the closest type strain was determined by RDP Seqmatch with the representative sequence of each OTU [76].

### Nucleotide Sequence Accession Numbers

The nucleotide sequences have been submitted to the European Nucleotide Archive under the accession number ERP001002.

## Supporting Information

Figure S1 Quantitative PCR-based detection of ^13^C-DNA in density gradient fractions (average from two independent measurements).
(TIF)

Table S1 Clusters (defined at 3% distance) of sequences detected in total community and ^13^C-DNA after 4- and 14-day incubation of soil with ^13^C-labeled substrates (biphenyl – Bp, benzoate – Bz, and naphthalene – Np). **Numbers of sequences in OTUs are normalized. Identification was performed by mothur-implemented RDP reference files** [**78**] **.**
(XLSX)

Table S2 Commands used for pyrosequencing data processing in mothur software package, version 1.25 [31], [72]. The table also summarizes the number of valid and unique sequences from a 10,000-read subset of mock community that passed the selected criteria.
(DOCX)
