# Supplementary material for: Identification of Bacteria Utilizing Biphenyl, Benzoate, and Naphthalene in Long-Term Contaminated Soil
Source: PLoS One. 2012 Jul 13;7(7):e40653. doi: 10.1371/journal.pone.0040653 (PMC3396604; doi:10.1371/journal.pone.0040653)
Supplement: Table S2 — Commands used for pyrosequencing data processing in mothur software package, version 1.25 [31], [72]. The table also summarizes the number of valid and unique sequences from a 10,000-read subset of mock community that passed the selected criteria. (DOCX) [file pone.0040653.s003.docx]

| **Step** | **Command** | **Parameters (**or for data summarizing number of valid and unique sequences in a 10,000-read mock community subset*)** |
| --- | --- | --- |
| Extracting sequences from a *sff* file | *sffinfo* | default |
| Trimming flowgram data | *trim.flows* | differences in the barcode: 1, differences in the primer: 2 |
| Denoising | *shhh.flows* | default |
| Primers removal, group file generation, quality screening | *trim.seqs* | differences in the barcode: 1, differences in the primer: 2, longest homopolymer: 8, minimum length: 200 |
| Data summarizing | *summary.seqs* | **** valid sequences: 8351, unique sequences: 5349*** |
| Unique sequences identification in a set | *unique.seqs* | default |
| Data summarizing | *summary.seqs* | **** valid sequences: 8351, unique sequences: 3777*** |
| Aligning sequences | *align.seqs* | reference: SILVA alignment |
| Data summarizing | *summary.seqs* | **** valid sequences: 8351, unique sequences: 3777*** |
| Removal of invalid sequences | *screen.seqs* | start: certain position of the alignment (primer f563-577 corresponds to alignment position 15,647), minimum length: based on information provided in the step above |
| Data summarizing | *summary.seqs* | **** valid sequences: 8308, unique sequences: 3734*** |
| Filtering positions out of an alignment | *filter.seqs* | removal of any column that contains exclusively gaps, removal of any column that has a "." character |
| Unique sequences identification in a set | *unique.seqs* | default |
| Removal of sequences that are likely due to pyrosequencing errors | *pre.cluster* | 1 difference per 100 bp (i.e. 2 differences for minimum length of 200 bp) |
| Data summarizing | *summary.seqs* | **** valid sequences: 8308, unique sequences: 876*** |
| Detection of chimeric sequences | *chimera.perseus* | default |
| Removal of chimeric sequences | *remove.seqs* | default |
| Data summarizing | *summary.seqs* | **** valid sequences: 5094, unique sequences: 181*** |
| Classifying sequences using RDP reference files | *classify.seqs* | cutoff: 50 (*recommended by RDP) |
| Data summarizing | *summary.seqs* | **** valid sequences: 5094, unique sequences: 181*** |
| Generation of a distance matrix | *dist.seqs* | cutoff: 0.15 |
| Clustering sequences into OTUs | *cluster* | default |
| Counting OTUs | *make.shared* | default |
| Classification of OTUs | *classify.otu* | distance: 0.03, cutoff > 50 |
| Removal of singletons, doubletons, and tripletons | *---* | **** 8 OTUs*** |
